# Determinants of anemia among pregnant mothers attending antenatal care in Dessie town health facilities, northern central Ethiopia, unmatched case -control study

**DOI:** 10.1371/journal.pone.0173173

**Published:** 2017-03-13

**Authors:** Sisay Eshete Tadesse, Omer Seid, Yemane G/Mariam, Abel Fekadu, Yitbarek Wasihun, Kedir Endris, Abebayehu Bitew

**Affiliations:** 1 Department of Nutrition, College Of Medicine and Health Science, Wollo University, Dessie, Ethiopia; 2 Department of Nutrition and Dietetics, College of Health Sciences, Mekelle University, Mekelle, Ethiopia; 3 Health Care Management, College of Health Sciences, Mekelle University, Mekelle, Ethiopia; 4 Department of Biostatics and Epidemiology, College Of Medicine and Health Science, University of Gondar, Gondar, Ethiopia; 5 Department of Health Education, College Of Medicine and Health Science, Wollo University, Dessie, Ethiopia; 6 Department of Nursing, College of Health Sciences, Mekelle University, Mekelle, Ethiopia; 7 Department of Biostatics and Epidemiology, College Of Medicine and Health Science, Debre Markos University, Debre Markos, Ethiopia; National Institute of Health, ITALY

## Abstract

**Introduction:**

Anemia affects around 38.2% and 22% of pregnant women at a global and national level respectively. In developing countries, women start pregnancy with already depleted body stores of iron and other vitamins with significant variation of anemia within and between regions.

**Objective:**

To identify the determinants of anemia among pregnant mothers attending antenatal care in Dessie town health facilities, northern central Ethiopia.

**Methods:**

A health facility based unmatched case control study was conducted among 112 cases and 336 controls from January to March 2016 G.C. The sample size was determined by using Epi Info version 7.1.5.2. Study subjects were selected using consecutive sampling technique. Data were collected using a structured questionnaire, entered using Epi Data version 3.1 and analyzed using SPSS version 20. Bivariable and multivariable logistic regression model was used to see the determinants of anemia. Adjusted odds ratio (AOR) with 95% confidence interval (CI) and p-value<0.05 were used to see the significant association.

**Results:**

Failure to take dark green leafy vegetables per two weeks (AOR = 5.02, 95% CI: 2.16, 11.71), didn’t take chicken per two weeks (AOR = 2.68, 95% CI: 1.22, 5.86), 1^st^ trimester (AOR = 2.07, 95% CI: 1.12, 3.84), 3^rd^ trimester (AOR = 2.96, 95% CI: 1.53, 5.72), HIV infection (AOR = 6.78, 95% CI: 2.28, 20.18) and medication (AOR = 3.57 95% CI: 1.60, 7.98) were positively associated with anemia.

**Conclusions:**

Inadequate intake of dark green leafy vegetables, inadequate consumption of chicken, trimester of the current pregnancy, HIV infection and medication were the determinants of anemia among pregnant women. Therefore, anemia prevention strategy should include promotion of adequate intake of dark green leafy vegetables and chicken, increase meal pattern during the entire pregnancy and strengthen the prevention of mother to child HIV transmission/antenatal care programs.

## Introduction

Globally anemia affects around 32.4 million (38.2%) of pregnant women. It is a severe public health problem in South East Asia (48.7%) and Africa (46.3%)[1]). The effect of anemia during pregnancy include less exercise tolerability, puerperal infection, thromboembolic problems, postpartum hemorrhage, pregnancy induced hypertension, placenta praevia, cardiac failure, low birth weight, preterm delivery, and prenatal death [2–4]. Worldwide it has been reported that nearly 510,000 maternal deaths occur per year associated with childbirth or early post-partum. Approximately 20% of maternal death is caused by anemia; the majority of this is taking place in developing countries [4].

In developing countries, pregnant women start pregnancy with already depleted body stores of iron and other vitamins. This is mainly due to poor nutritional intake, repeated infections, menstrual blood loss and frequent pregnancies. It is also associated with socioeconomic conditions, lifestyles, and health-seeking behaviors across different cultures [2,3].

Despite the efforts made by the government and other stakeholders, anemia during pregnancy is still a public health problem in Ethiopia. There is a significant variation in the prevalence of anemia within and between regions. Even though many researches have been conducted using cross sectional study, their result revealed that determinants of anemia were vary from place to place [4–8]. This indicates a need for local data to address the problem. In addition, the nature of cross sectional study design makes them weak in showing the real causal association between anemia and its determinants among pregnant women. In spite of probing efforts, in the study area, factors associated with anemia among pregnant mothers are limited or non-existent. Therefore, this study has addressed the identified gaps and examined the determinants of anemia using case control study among pregnant women attending antenatal care in health facilities of Dessie town.

## Methods

### Study area, study period and study population

The study was conducted from January to March 2016 G.C among pregnant women attending ANC in selected health facilities of Dessie town. Dessie is one of the towns found in Amhara national regional state, in northern central Ethiopia. It is located at 401 km from the capital of Ethiopia, Addis Ababa. According to the city administrative health office the total population of the town is 218,471. About 102,378 (46.86%) are males and 116,093 (53.14%) are females. The town is found at an altitude of 2470 to 2550 meters above sea level. Currently the town has five hospitals (one public referral, one public general and three private), eight health centers and twenty seven private clinics.

### Study design

A health facility based unmatched case control study design was employed.

### Inclusion and exclusion criteria

Pregnant women who attend ANC in health facilities during their 1^st^ visit and those who are residents for a minimum of six months in the town were included in the study. While, those who were severely ill and those with 2^nd^ and 3^rd^ visits were excluded from the study.

### Sample size determination

The sample size was calculated using Epi Info version 7.1.5.2 by considering the following assumptions. The expected frequency of intestinal parasite among anemic mothers in Ethiopia was 41.7% [4], 95% CI, 80% power and a ratio of 1:3. Thus, considering 10% non-response rate, the total number of study participants was 448.

### Sampling technique

There are 17 health facilities that provide ANC service in Dessie town. Of these, six were randomly selected and included in the study. Numbers of study participants were assigned to each selected health facility proportional to their average client size attended per month by referring the registration books of each antenatal care unit. Thus, mathematically, average number of pregnant women who attended antenatal care in each health facilities per month multiplied by the total sample size (N = 448), divided by the total number of pregnant women attended in the entire antenatal care unit per month (270). Finally the study subjects were drawn from each selected health facility using consecutive sampling technique.

### Standard definition

#### Anemia

Hemoglobin cutoff value adjusted at sea level altitude was used to define anemia on the basis of gestational age using WHO criteria. The Hgb value less than 11.0 g/dl at 1^st^ and 3^rd^ trimesters and less than 10.5 g/dl at 2^nd^ trimester were used to define anemia [9].

### Data collection procedure

Data were collected by face to face interview using a structured questionnaire adapted from previous similar literatures. Blood hemoglobin was determined using hemocue. Data collectors were nurses and midwiferies. The questionnaire gathered five groups of participants’ characteristics, namely socio-demographic and economic, disease, gynecological and obstetric, dietary habit and nutrition, and WASH related factors. A stool sample was collected from each study participant using clean, wide mouthed and leak proof stool cup. Then, stool wets mount was prepared using saline and/or iodine and examined microscopically for identification of intestinal helminthes and protozoan parasites within 30 minutes of collection. Serum and/or plasma sample was tested for HIV following the current HIV1/2 testing algorithm.

Dietary Diversity Score (DDS) was calculated from a single 24 hour dietary recall data. All the foods and the liquids consumed a day before the study was categorized into 9 food groups. Consuming a food item from any of the groups was assigned a score of 1 and if no food was taken a score of 0 was given. Accordingly, a DDS of 9 points was computed by adding the values of all the groups. Then it was categorized as low (≤3), medium (4–6) and high (7–9) [10]. Additionally, study participants were screened for nutritional status using MUAC measuring tape.

### Data quality assurance

The questionnaire was translated to Amharic and back to English for consistency. Pre-test was conducted at 5% of the sample size in Boru Meda general hospital. Three day intensive training was given for data collectors and a supervisor on the overall data collection procedure to minimize systematic error. On spot checking and correction was made for incomplete questionnaire by supervisor. The overall data collection process was controlled by the principal investigator. Laboratory test quality was assured by giving training for laboratory professionals, using standard operating procedure (SOP) and regular monitoring of reagents for manufacturing, expiry date and proper storage. The sample was processed immediately after collecting from the study participant to minimize errors.

### Data analysis

After coding the questionnaire the data were entered into Epi Data version 3.1 for cleaning and exported to SPSS version 20.0 for analysis. Outcome variable was dichotomized into 1 = cases and 0 = controls. First, descriptive statistics were computed and the result was reported using frequencies and percentages. Next bivariate logistic regression was performed and variables with p < 0.25 were transported to multivariable logistic regression to identify the determinants of anemia among pregnant mother. Finally, variables with P-value < 0.05 in the multivariable logistic regression model were taken as statistically significant and adjusted odds ratio with its 95% confidence interval was considered to see the association. Multicollinearity test was done using variance inflation factor (VIF) and no collinearity exists between the independent variables. The model goodness of the test was checked by Hosmer- Lemeshow goodness of the fit and the p-value of the model fitness of the test was 0.78.

### Ethical consideration

The proposal was reviewed and approved by the Institutional Review Board (IRB) of Mekelle University College of Health Sciences. Written permission was obtained from Dessie city administrative health department and selected health institutions. After the purpose and objectives of the study have been informed, written consent was obtained from each study participant. Written assent was obtained for those whose age was below 18 years and above from their kin. Participants were informed as participation was on a voluntary basis. The data collection procedure was anonymous for keeping the confidentiality of any information. Those anemic cases were given iron folic acid and counseled to take iron rich foods.

## Results

### Socio demographic and economic factors

Four hundred forty eight pregnant women with 112 cases and 336 controls were recruited in this study. The response rate was 100%. The age of study participant ranged from 17–38 years of age. From these above one third 174 (38.8%) were found in the age range of 25–29 years. Forty seven (42%) of the cases and 127 (37.8%) of the controls were found between 25–29 years. The average monthly family income of slightly one third of the cases falls between 1500–2499 Birr (39.1%) as compared to the control group (25.3%) (Table 1).

**Table 1:** Socio-demographic and economic characteristics of pregnant mother attending ANC in health facilities of Dessie town, from January to March 2016 G.C.

Variables		Case: n = 112 (%)	Control: n = 336 (%)
Ethnicity	Amhara	108(96.4%)	326(97.0%)
	Others^a^	4(3.6%)	10(3%)
Age group	15–19	3(2.7%)	15(4.5%)
	20–24	37(33%)	127(37.8%)
	25–29	47(42%)	127(37.8%)
	30–34	22(19.6%)	54(16.1%)
	35–39	3(2.7%)	13(3.9%)
Religion	Orthodox	38(33.9%)	117(34.8%)
	Muslim	71(63.4%)	213(63.4%)
	Others^b^	3(2.7%)	6(1.8%)
Formal Educational (Mother’s)	No	24(21.4%)	39(11.6%)
	Yes	88(78.6%)	297(87.4%)
Formal Education (Husband’s)	No	17(15.3%)	32(9.8%)
	Yes	94(84.6%)	296(90.2%)
Family Monthly Income	≤1499	19(17%)	50(14.9%)
	1500–2499	38(33.9%)	85(25.3%)
	2500–3499	31(27.7%)	113(33.6%)
	> = 3500	24(21.4%)	88(26.2%)
Occupation	Employed	22(19.6%)	84(25%)
	Farmer	22(19.6%)	27(8%)
	Housewife	45(40.2%)	139(41.4%)
	Private work	22(19.6%)	79(23.5%)
	Other^c^	1(0.9%)	7(2.1%)

^a^- Tigre, Oromo and Gurage,

^b^- Catholic and protestant,

^c^- Student.

### Hygiene and sanitation related factors

The majority of the cases 106 (94.6%) and almost all of the controls 334 (99.4%) were using tap water for drinking. Almost all 111 (99.1%) of the cases and 333 (99.1%) of the controls were having latrine. Regarding the hand washing practice, 99 (88.4%) of the cases and 325 (96.7%) of the controls were practicing hand washing before meal.

### Dietary habit and nutrition related factors

More than three fourth 94 (83.9%) of the cases and majority 312 (92.9%) of the controls were using teff Injera and wot (Ethiopian stew) as a staple food. Only 7 (6.2%) of the cases and 33 (9.8%) of the controls were having a meal frequency of less than twice per day and the rest was eating foods three times and above. About 16 (14.3%) of the cases and 79 (23.5%) of the controls were eating DGLV daily, while 29 (25.9%) of the cases and 27 (8.0%) of the controls were taking DGLV once per two weeks. Almost two third 72 (64.3%) of the cases and more than one third 126 (37.5%) the controls didn’t consume red meat per two weeks. Nearly one fourth 27 (24.1%) of the cases and 144 (42.9%) of the controls were eating red meat once per weeks. About 10 (8.9%) of the cases and 74 (22%) of the controls were eating chicken once per two weeks, while the rest from both groups didn’t take chicken. One fourth 28 (25%) of the cases and one third 108 (32.1%) of the controls were having a MUAC < 23 cm. Two third of the cases 75 (67%) and 212 (63.1%) of the controls had a medium (4–6) dietary diversity score. More than one third 39 (34.8%) of the cases and 146 (43.5%) of the controls had an appetite loss during their pregnancy.

### Disease related factors

Slightly lower than a quarter 25 (22.3%) of the cases and 21 (6.2%) of the controls was using medication during their pregnancy. The commonest drugs taken by study participants were gastritis drugs (52%), ARV drugs (30.4%)) and anti-TB drugs (4.3%). Sixteen (14.3%) of the cases and 8 (2.4%) of the controls were reactive for HIV. One fourth 28 (25%) of the cases and 73 (21.7%) of the controls were having gastritis/PUD. Majority 99 (88.4%) of the cases and 316 (94%) of the controls had no intestinal parasite and the rest from both groups were having amoeba, bacteria and Ascaris.

### Determinant of anemia

Crude odds ratio was performed for each independent variable. Variables having a p-value of less than 0.25 were transferred to multivariable logistic regression. The result in multivariable logistic regression showed that the odds of getting anemia in pregnant mothers who didn’t consume dark green leafy vegetables were five times greater than the odds of mothers who consume on a daily basis (AOR = 5.02, 95% CI: 2.16,11.71). This study signifies that pregnant women who didn’t take chicken were 2.7 times more likely to have a risk of developing anemia than who took once per two weeks (AOR = 2.68, 95% CI: 1.22, 5.86).

According to this study pregnant mothers who were in the first trimester were almost two times more likely to develop anemia than those in the second trimester (AOR = 2. 07, 95% CI: 1.12, 3.84). Similarly, pregnant mothers in the third trimester were almost three times more likely to be anemic than those in the second trimester (AOR = 2. 96, 95% CI: 1.53, 5.72). The odds of developing anemia among HIV reactive pregnant women were 6.8 times higher than HIV non-reactive pregnant mother (AOR = 6.78, 95% CI: 2.28, 20.18). In this study medication was also found to be a significant predictor of anemia. Those mothers who take medication were 3.6 times more likely to develop anemia than those who didn’t take any medication (AOR = 3.57, 95% CI: 1.60, 7.98) (Table 2).

**Table 2:** Determinants of anemia among pregnant mothers attending ANC in health facilities of Dessie town, January to March 2016 G.C.

Variables		Cases	Controls	COR (95% CI)	AOR (95% CI)
Frequency of DGLV	Daily	16(14.3%)	79(23.5%)	1	1
	Every other day	11(9.8%)	73(21.7%)	0.74(0.32,1.71)	0.83(0.31,2.11)
	1–2 times/weeks	46(41.1%)	142(42.3%)	1.59(0.85,3.01)	1.64(0.81,3.30)
	Once/2 weeks	29(25.9%)	27(8.0%)	5.30 (2.50,11.23)	4.89(1.68, 14.24)
	Don’t take	10(8.9%)	15(4.5%)	3.29(1.26,8.63)	5.02 (2.16,11.71)
Frequency of Chicken	Once/2 weeks	10(8.9%)	74(22%)	1	1
	Don’t take	102(91.1%)	262(78.0%)	2.88 (1.43,5.79)	2.68(1.22, 5.86)
Duration of menstruation	≤3 days	22(19.6%)	91(27.1%)	1	
	4–7 days	72(64.3%)	221(65.8%)	1.35(0.79,2.30)	
	≥8 days	18(16.1%)	24(7.1%)	3.1(1.44,6.69)	
Gestational Age	1^st^ trimester	27(42.1%)	63(18.8%)	1.69(0.99,2.87)	2.07 (1.12,3.84)
	2^nd^ trimester	59(52.7%)	232(69%)	1	1
	3^rd^ trimester	26(23.2%)	41(12.2%)	2.49 (1.41,4.40)	2.96 (1.53,5.72)
Presence of chronic disease	Yes	19(17%)	19(5.7%)	3.41(1.73,6.71)	
	No	93(83%)	317(94.3%)	1	
HIV Test	Reactive	16(14.3%)	8(2.4%)	6.83(2.84,16.45)	6.78(2.28,20.18)
	Non-Reactive	96(85.7%)	328(97.6%)	1	1
Medication	Yes	25(22.3%)	21(6.2%)	4.31(2.30,8.07)	3.57(1.60, 7.98)
	No	87(77.7%)	315(93.8%)	1	1

## Discussion

Anemia is a worldwide, including Ethiopia, public health problem which increases the risk of maternal and child morbidity and mortality, impaired cognitive and physical development of children and decrease work productivity in adults [11]. Prevention and control of anemia among pregnant women are therefore a key measure to reduce the adverse effects of anemia, which will help to have healthy and productive future generation. So identifying the determinant of anemia is an input to take evidence based interventions.

Therefore, among the main determinants identified in this study, inadequate intake of DGLV was appeared to be positively related to anemia development among pregnant mothers. Similarly, other literatures showed that lower consumption of DGLV was significantly associated with increased risk of developing anemia [3,8,12]. This might be because low consumption of DGLV results in reduced intake of non-heme iron, vitamin A and vitamin C. these vitamins (vitamin A and C) have an absorption promoting effect on non-heme iron [13]. Thus, their deficiency could be another substantiating factor for the existence of iron deficiency anemia (IDA). In contrast, studies from Eastern Ethiopia and East Anatolian Province (Turkey) reported that intake of DGLV had no association with anemia [14]. This varying result might be due to other components of DGLV that may have either an enhancing or inhibiting effect on iron absorption and the level of body iron stores of the study participants.

Another interesting finding of this study is that inadequate intake of chicken was significantly associated with anemia. Other literatures revealed that consumption of heme iron rich food sources (chicken) were associated with lower prevalence of anemia [7,15]. This could be because chicken is a rich source of heme iron, which is highly bioavailable and has an enhancing effect on non-heme iron absorption. Therefore, inadequate intake of chicken has the possibility to increase the occurrence of IDA [16]. This finding strengthens the report of WHO, which stated that in developing countries inadequate intake of dietary iron is the main causes of anemia during pregnancy [13].

The risk of developing anemia was more likely higher in the 1^st^ and 3^rd^ trimester of pregnancy. Taking the second trimester as a reference group, the result of multivariable logistic regression analysis signifies that pregnant mothers who were in the first and third trimesters were significantly associated with the development of anemia and the odds is higher in the third trimester. The possible justification, first trimester, could be due to loss of appetite, morning sickness and the start of hemodilution at 8 weeks of gestation. While in the 3^rd^ trimester it might be because of the high nutrient requirement for the growing fetus and sharing of iron in the blood to the fetus which will reduce the maternal iron reserves. This finding is consistent with a study done in West Arsi zone Oromia Ethiopia, East Anatolian Province (Turkey) and South Eastern Nigeria, which revealed that the prevalence of anemia is higher in the third trimester of pregnancy [3,8,17]. But evidences from Southeast Ethiopia showed that gestational age had no association with the occurrence of anemia [4]. The difference might be due to variation in methods.

An important association was seen between anemia and HIV/AIDS infection. Those pregnant mothers with HIV positive were 6.8 times more likely to have a risk of developing anemia than their counterparts. This finding was similar with previous findings from North East Ethiopia, South West Nigeria and South East Nigeria [14,18,19]. This could be as a result of the enhancement of nutritional deficiencies, suppression of bone marrow due to cytokine production [20]; opportunistic infection and use of antiretroviral drugs in patients with AIDS which interfere with deregulation of the host immune system leading to destruction or inhibition of hematopoietic cells [21].

This study also showed that intake of drugs has a strong positive significant association with the occurrence of anemia among pregnant women. These drugs were supposed to cause anemia either by interfering with the absorption of micronutrients or inhibiting the effect of erythropoietin that is important for hemoglobin production[22,23].

As far as the study design was a retrospective in nature, it may be affected by recall bias. Being a facility based study and seasonal variation of dietary intake could also be other limitations of this study.

To conclude, inadequate intake of DGLV, no consumption of chicken, trimester of the current pregnancy, HIV positive and medication were positively associated with anemia development among pregnant women. Therefore, anemia prevention strategy should include promotion of adequate intake of DGLV, intake of chicken, increase meal pattern during the entire trimester and strengthen PMTCT/ANC programs.

## Supporting information

S1 File Questionnaire used for data collection. (DOCX)

## Abbreviations

95% CI: 95% Confidence Interval
AIDS: Acquired Immuno Deficiency Syndrome
ANC: Antenatal Care
AOR: Adjusted odds ratio
ARV: Anti-retroviral
COR: Both Crude Odds Ratio
DGLV: Dark Green Leafy Vegetables
HIV: Human Immunodeficiency Virus
IDA: Iron Deficiency Anemia
MUAC: Mid-Upper Arm Circumference
PMTCT: Prevention of Mother to Child Transmission
PUD: Peptic Ulcer Disease
SOP: Standard Operating procedure
SPSS: Statistical package for social sciences
TB: Tuberculosis
WHO: World Health Organization
