# Supplementary material for: Determinants of anemia among pregnant mothers attending antenatal care in Dessie town health facilities, northern central Ethiopia, unmatched case -control study
Source: PLoS One. 2017 Mar 13;12(3):e0173173. doi: 10.1371/journal.pone.0173173 (PMC5348124; doi:10.1371/journal.pone.0173173)
Supplement: S1 File — (DOCX) [file pone.0173173.s001.docx]

S1 file: Questionnaire used for data collection

| Questionnaires ID.No……… Address……..... Name of health facility............ Date of interview……………… Time started……… Time finished………. Code number………: | | | |
| --- | --- | --- | --- |
| S.No | **Questionnaire** | **Code** | **Skip** |
| **Part I: Questions on socio-demographic and economic characteristics** | | |  |
| 101 | Age of mother in complete year: ……………… | Kebele or sub city ……… |  |
| 102 | Religion: | 1. Orthodox 2. Muslim 3. Catholic 4. Protestant 5. Others: ……………… |  |
| 103 | Ethnicity | 1. Amhara 2. Tigray 3. Oromo 4. Other: ……………… |  |
| 104 | How many family members are living with you? ……………… | |  |
| 105 | What is the highest educational level you have completed? | 1. Illiterate 2. Elementary 3. High school 4. Diploma 5. 1^st^ degree & above |  |
| 106 | What is your current marital status? | 1. Single 2. Married 3. Widowed 4. Divorced |  |
| 107 | What is the highest educational level your current husband has completed? | 1. Illiterate 2. Elementary 3. High school 4. Diploma 5. 1^st^ degree & above | Skip it if not currently married |
| 108 | Mather’s Occupation | 1. Civil servant 2. Agriculture/farmer 3. Own business runner 4. Wage work permanently 5. Wage work occasionally 6. Merchant 7. Housewife 8. Retired 9. Other: ……………… |  |
| 109 | What is your family monthly income? …………… Birr | |  |
| **Part II: Questions on hygiene and sanitation related factors** | | | |
| 201 | What is your main source of water for drinking? | 1. Tap water 2. River water 3. Protected well 4. Unprotected well 5. Other……………… |  |
| 202 | Do you have latrine? | 1. Yes 2. No | If no, go to Q_204_ |
| 203 | If yes to #Q_202_ did you use it appropriately? | 1. Yes 2. No |  |
| 204 | When did you wash your hands? (More than one option is possible) | 1. Before meal 2. After using the toilet 3. After having a meal 4. Other……………… |  |
| **Part III: Questions on dietary habit related factors** | | | |
| 301 | What are your staple foods? | 1. Teff Injera and wet 2. Maize and Sorghum 3. Spaghetti and Rice 4. Vegetables/ fruits 5. Other: …………… |  |
| 302 | How many times per day did you eat yesterday? | 1. Twice 2. Three times 3. Four times 4. Five times and above |  |
| 303 | Was there any foods forbidden for pregnant mothers in your culture? | 1. Yes 2. No | If no, go to Q_305_ |
| 304 | If yes to #Q_303_ Please mention them  ………………………………………………………………………………… | |  |
| 305 | Why they are forbidden? …………………………………………………… | |  |
| 306 | Did you fast during your pregnancy? | 1. Yes 2. No |  |
| 307 | What is the status of your appetite (eating condition) during pregnancy? | 1. Decreased 2. Increased 3. No change |  |
| 308 | Did you use tea/coffee immediately after having a meal? | 1. Yes  2. No |  |
| **Part IV: Questions on gynecological and obstetric related factors** | | |  |
| 401 | Have you ever been experienced with abortion? | 1. Yes 2. No | If no, go to Q_403_ |
| 402 | If yes, for #Q_401_, how many times? | …………… Times |  |
| 403 | Was your menstruation coming regularly before the index pregnancy? | 1. Yes  2. No |  |
| 404 | For how many days your menstruation lasts? …………… | |  |
| 405 | Can you tell me the rate of flow of your menstruation during its period? | 1. Very heavy 2. Heavy 3. Moderate 4. Low |  |
| 406 | Is it your first pregnancy? | 1. Yes 2. No | If yes, go to Q_412_ |
| 407 | If no to #Q_406_ how many times have you been pregnant? | 1. Twice 2. Three times 3. 4 times 4. Other…………… |  |
| 408 | How long was the interval between your previous and current pregnancy? | …… Year(s)…..... Months (>2 pregnancies) |  |
| 409 | How long was the interval between your first and second pregnancy? | …… Year…… Months (> 3 pregnancies) |  |
| 410 | How long was the interval between your second and third pregnancy? | …...Year(s)…… months  (> 4 pregnancies) |  |
| 411 | Please indicate the intervals between your rest pregnancies. | …….....………….....…………....……………… |  |
| 412 | Do you have a virginal bleeding during this pregnancy? | 1. Yes  2. No |  |
| 413 | Have you ever used a family planning method before you are pregnant? | 1. Yes 2. No | If no, go to Q_417_ |
| 414 | If yes to #Q_412_ Which methods did you use? | 1. Pills 2. Injectable 3. IUCD 4. Implant |  |
| 415 | For how many years did you use? | 1. Less than 1 2. 1-2 Yrs 3. 2-4 Yrs 4. More than 4 yrs |  |
| 416 | Did it have any change in your menstrual flow? | 1. Yes 2. No |  |
| 417 | If yes to #Q_415_ please explain the occasion  In terms of flow………….....…………..  In terms of regularity………….....…… | |  |
| **Part V: Questions on disease related factors** | | | |
| 501 | Did you take any medication? | 1. Yes  2. No |  |
| 502 | If yes to #Q_418_ for what disease are you taking it? ………….....………….. | |  |
| 503 | Did you have a history of medical surgery? | 1. Yes 2. No |  |

**Part VI: Clinical findings by examination**

| 504 | Presence of chronic disorders | 1. Hypertension 2. Diabetes 3. TB 4. Other…….....……… |  |
| --- | --- | --- | --- |
| 505 | Presence of gastritis/peptic ulcer disease | 1. Yes 2. No |  |

**Part VII: Laboratory test results record format**

| 601 | Stool Examination | 1. No parasite seen 2. Hookworm 3. Ascaris 4. Schistosoma 5. …….....……… |  |
| --- | --- | --- | --- |
| 602 | Hemoglobin level | ……..…….. |  |
| 603 | HIV Sero-positivity | 1. Reactive 2. Non-reactive |  |

MUAC…….. Gestational age……..

**Table 3: Tools used for laboratory diagnosis**

| **Type of test** | **Equipment used** |
| --- | --- |
| Hemoglobin level | Hemocue, micro cuvette, Lancet, Alcohol and Cotton |
| Stool examination | Slide, Cover slide, Applicator Stick, Saline, Stool Container, Microscope |
| HIV Sero-positivity test | KHB, stat pack Uni-gold, lancet, EDA Capillary tube, Cotton, Alcohol and timer, |

## **Dietary diversity questionnaire formats**

Please describe the foods (meals and snacks) that you ate or drank yesterday during the day and night, whether at home or outside the home. Start with the first food or drink eaten in the morning.

Write down all food and drinks mentioned. When composite dishes are mentioned, ask for the list of ingredients. When the respondent has finished, probe for meals and snacks not mentioned.

**Part IX: Format for conducting dietary intake using 24 hour recall**

| **Breakfast** | **Snack** | **Lunch** | **Snack** | **Dinner** | **Snack** |
| --- | --- | --- | --- | --- | --- |
|  |  |  |  |  |  |

Did you eat anything (a meal or snack) OUTSIDE the home yesterday? When the respondents recall is complete, fill in the food groups based on the information recorded above. For any food groups not mentioned, ask the respondent if a food item from this group was consumed.

**Part X: Format for summarized women’s dietary diversity score**

| **Starchy Samples** | **Vit A rich**  **Fruits/Vegetables** | **Other Fruits/**  **Vegetables** | **Dark green leafy vegetables** | **Organ**  **meat** | **Flesh**  **meat** | **Eggs** | **Legumes/ Nuts** | **Milk** | **WDDS** |
| --- | --- | --- | --- | --- | --- | --- | --- | --- | --- |
|  |  |  |  |  |  |  |  |  |  |

**Part XI: Food frequency questionnaire**

| **List of foods** | **Frequency of consumption** | | | | |
| --- | --- | --- | --- | --- | --- |
|  | **Daily** | **Every other day** | **1-2 times in a week** | **Once in two weeks** | **Don't take** |
| Red meat |  |  |  |  |  |
| Organ meat (Liver, kidney, etc.) |  |  |  |  |  |
| Dark green leafy vegetable (Lettuce, cabbage, pepper) |  |  |  |  |  |
| Fruits (mango, avocado, papaya, lemon, orange, etc.) |  |  |  |  |  |
| Legumes (peas, beans, lentils, soy beans etc.) |  |  |  |  |  |
| Eggs |  |  |  |  |  |
| Chicken |  |  |  |  |  |
| Fish |  |  |  |  |  |
| Food made from teff |  |  |  |  |  |
| Milk & milk product |  |  |  |  |  |

Interviewer Name………………..…….. Date……………… Signature: ………………

**Table 5: Annex 2: Amharic version questionnaire**

| የመጠይቁ መ.ቁ…… አድራሻ ……... የጤና ተቋሙ ስም ............ ቃለ መጠይቅ የተደረገበት ቀን ……………… የተጀመረበት ሰዓት……… ያለቀበት ሰዓት………. ኮድ ………. | | | | | |
| --- | --- | --- | --- | --- | --- |
| ተ.ቁ | | **ቃለ መጠይቅ** | | መለያ ቁጥር | ዝለል |
| **ክፍል አንድ : ሥነ-ሕዝብና ኢኮኖሚን የተመለከተ ጥያቄ** | | | | |  |
| 101 | | የእናት አድሜ: ……………… | ቀበሌ/ ክ/ከ …………… | |  |
| 102 | | ሃይማኖትሽ ምንድን ነው? | 1. ኦርቶዶክስ 2. ሙስሊም 3. ካቶሊክ 4. ፕሮቴስታንት 5. ሌላ: ……………… | |  |
| 103 | | የቤተሰብሽ ብዛት ስንት ነው?……………… | | |  |
| 104 | | ብሔርሽ ምንድን ነው? | - 1. አማራ   2. ትግሬ   3. ኦሮሞ   4. ሌላ:………… | |  |
| 105 | | የጋብቻ ሁኔታ | 1. ያላገባች 2. ያገባች 3. ባሏ የሞተባት 4. የተፋታች | |  |
| 106 | | የትምህርት ደረጃሽ ስንት ነው? | 1. ያልተማረች 2. 1^ኛ^ ደረጃ 3. 2^ኛ^ ደረጃ 4. ዲፕሎማ 5. 1^ኛ^ ዲግሪና በላይ | |  |
| 107 | | የባልሽ የትምህርት ደረጃ ስንት ነው? | 1. ያልተማረ 2. 1^ኛ^ ደረጃ 3. 2^ኛ^ ደረጃ 4. ዲፕሎማ 5. 1^ኛ^ ዲግሪና በላይ | |  |
| 108 | | ስራሽ ምንድን ነው? | 1. መንግስት ሰራተኛ 2. ገበሬ 3. የግል ስራ ያላት 4. ቋሚ የቀን ሰራተኛ 5. ጊዜያዊ የቀን ሰራተኛ 6. ነጋዴ 7. የቤት እመቤት 8. ጡረተኛ 9. ሌላ: ……………… | |  |
| 109 | | የቤተሰብሽ ወርሃዊ ገቢ ስንት ነው?: …………… ብር | | |  |
| **ክፍል ሁለት: የግልና የውሃ ንፅህና** | | | | | |
| 201 | | ለመጠጥ የሚሆን ውሃ የምታገኙት ከየት ነው? | 1. ከቧንቧ 2. ከወንዝ ውሃ 3. ከተጠበቀ ጉድጓድ 4. ካልተጠበቀ ጉድጓድ 5. ሌላ ……………… | |  |
| 202 | | መጸዳጃ ቤት አላችሁ? | 1. አዎ 2. የለኝም | | የለም ከሆነ ወደጥ_204_ |
| 203 | | በትክክል ትጠቀሙበታላችሁ? | 1. አዎ  2. የለኝም | |  |
| 204 | | እጅሽን መቼ ትታጠቢያለሽ? | 1. ከምግብ በፊት 2. ከምግብ በኋላ 3. ከመፀዳጃ መልስ 4. ሌላ …………… | |  |
| **ክፍል ሶስት: የእናት አመጋገብን የተመለከቱ ጥያቄዎች** | | | | | |
| 301 | | አዝወትረሽ የምትመገቢው የምግብ ዓይነት ምንድን ነው? | 1. የጤፍ እንጀራ በወጥ 2. ከበቆሎና ማሽላ የተዘጋጀ ምግብ 3. ፓስታና ሩዝ 4. አትክልትና ፍራፈሬ 5. ሌላ: …………… | |  |
| 302 | | በእርግዝናሽ ወቅት በቀን ስንቴ ትመገቢያለሽ? | 1. ሁለት ጊዜ 2. ሶስት ጊዜ 3. አራት ጊዜ 4. አምሰት ጊዜ | |  |
| 303 | | በባህላችሁ ለእርጉዝ እናት የሚከለከል ምግብ አለ? | 1. አዎ 2. የለም | | የለም ከሆነ ወደጥ_306 ሂድ_ |
| 304 | | ለጥያቄ ቁ_303_ መልስሽ አዎ ከሆነ የሚከለከሉትን ምግቦች ዘርዝሪ ………………………………………………………………………… | | |  |
| 305 | | ለምን ይከለከላሉ? …………………………………………………………………………… | | |  |
| 306 | | በእርግዝናሽ ወቅት ትፆሚያለሽ? | - 1. አዎ   2. የለም | |  |
| 307 | | ለጥያቄ ቁ_306_ መልስሽ አዎ ከሆነ ምን ምን ትፆሚያለሽ? | 1. ከእንስሳት ተዋፅኦ ብቻ 2. ከእንስሳት ተዋፅኦና የፆም ምግብ እስከ ስድስት ሰዓት 3. ሌላ፡………………… | |  |
| 308 | | በእርግዝናሽ ወቅት (በትላንትናው ዕለት) የምግብ ፍላጎትሽ እንዴት ነው? | 1. ይቀንሳል 2. ይጨምራል 3. ለውጥ የለውም | |  |
| 309 | | በእርግዝናሽ ወቅት የሚያምርሽ ነገር አለ? | 1. አዎ 2. የለም | | የለም ከሆነ ወደጥ_311 ሂድ_ |
| 310 | | ካለ የሚያምርሽን ዘርዝረሽ አስረጂኝ ………………………………… | | |  |
| 311 | | ሻይ ወይም ቡና ከምግብ ጋር ወይም ወዲያው ከምግብ ቀጥሎ ትጠቀሚያሽ? | 1. አዎ 2. የለም | |  |
| **ክፍል አራት: ማህፀንንና ጽንስን (ጋይኒ ኦብስን) የተመለከቱ ጥያቄዎች** | | | | |  |
| 401 | ውርጃ አጋጥሞሽ ያውቃል? | | 1. አዎ 2. የልም | |  |
| 402 | አዎ ከሆነ ስንቴ ገጥሞሽ ያውቃል? …………… | | | |  |
| 403 | ከማርገዝሽ በፊት የወር አበባሽ በወቅቱ ይመጣል? | | 1. አዎ 2. የለም | |  |
| 404 | የወር አበባሽ ለስንት ቀን ይቆያል? …………… | | | |  |
| 405 | በወር አበባሽ ጊዜ የሚፈሰው የደም መጠን ብዛቱ እንዴት ነው? | | 1. በጣም ቢዙ ነው 2. ብዙ ነው 3. መካከለኛ ነው 4. ትንሽ ነው | |  |
| 406 | እርግዝናው የመጀመሪያሽ ነው? | | 1. አዎ 2. የለም | | አዎ ከሆነ ወደጥ_412 ሂድ_ |
| 407 | ለጥያቄ ቁ_406_ መልስሽ የለም ከሆነ እርግዝናው ስንተኛሽ ነው? | | 1. ሁለተኛ 2. ሶስተኛ 3. አራተኛ 4. ሌላ …………… | |  |
| 408 | የባለፈውና የአሁኑ እርግዝና ምን ያህል ይራራቃሉ? | | …… ዓመት …..... ወር (ሁለትና ከዚያ በላይ ለሆነ) | |  |
| 409 | የመጀመሪያውና የሁለተኛው እርግዝና ምን ያህል ይራራቃሉ? | | …… ዓመት …..... ወር (ሶስትና ከዚያ በላይ ለሆነ) | |  |
| 410 | የሁለተኛውና የሶስተኛው እርግዝና ምን ያህል ይራራቃሉ? | | …… ዓመት …..... ወር (አራትና ከዚያ በላይ ለሆነ) | |  |
| 411 | እባክሽ በቀሪዎቹ እርግዝናዎች መካከል ያለውን የጊዜ ልዩነት ንገሪኝ? | | …….....………….....…………....……………… | |  |
| 412 | እርጉዝ ከመሆንሽ በፊት የቤተሰብ ዕቅድ ተጠቃሚ ነበርሽ? | | 1. አዎ 2. የለም | | የለም ከሆነ ወደጥ_417 ሂድ_ |
| 413 | ለጥያቄ ቁ_412_ መልስሽ አዎ ከሆነ የትኛውን ዓይነት ነው የተጠቀምሽው? | | 1. ኪኒን 2. መርፌ 3. በማኅፀን የሚቀበረውን 4. በክንድ ስር የሚቀበረውን | |  |
| 414 | ለስንት ዓመት ተጠቀምሽ? | | 1. ለአንድ ዓመት 2. 1-2 ዓመት 3. 2-4 ዓመት 4. ከ 4 ዓመት በላይ | |  |
| 415 | ስትጠቀሚ በወር አበባሽ ላይ ለውጥ ነበረው? | | 1. አዎ 2. የለም | | የለም ከሆነ ወደጥ_417 ሂድ_ |
| 416 | ለጥያቄ ቁ_415_ መልስሽ አዎ ከሆነ በሚከተለው መሰረት አብራሪው  የሚፈሰው መጠን ………….....…………..  በወቅቱ ይመጣ ነበር? ………….....…… | | | |  |
| 417 | **ክፍል አምስት: በሽታን የተመለከቱ ጥያቄዎች** | | | | |
| 418 | የጨጓራ/የአንጅት ቁስለት በሽታ አሞሽ ያውቃል? | | 1. አዎ 2. የለም | |  |
| 419 | በአሁኑ ሰዓት መድኃኒት እየወሰድሽ ነው? | | 1. አዎ 2. የለም | |  |
| 420 | መልስሽ አዎ ከሆነ የምን መድኃኒት እየወሰድሽ ነው? | | ………….....………….....………….....…… | |  |
| 421 | ስር የሰደደ በሽታ አሞሽ ያውቃል? | | 1. አዎ 2. የለም | |  |
| 422 | መልስሽ አዎ ከሆነ ከተዘረዘሩት ውስጥ በየትኛው ነው የታመምሽው? | | 1. ስር የሰደደ የኩላሊት በሽታ 2. ኤች.አይ.ቪ 3. የሳንባ በሽታ 4. የጉበት በሽታ 5. የደም ግፊት 6. ሌላ …….....……… | | 2 ወደ ጥ_43_ሂድ |
| 423 | ከአሁን በፊት ቀዶ ህክምና ተሰርቶልሽ ያውቃል? | | 1. አዎ 2. የለም | |  |
